# How was research engaged with and used in the development of 131 policy documents? Findings and measurement implications from a mixed methods study

**DOI:** 10.1186/s13012-019-0886-2

**Published:** 2019-04-30

**Authors:** Anna Williamson, Steve R. Makkar, Sally Redman

**Affiliations:** 1 The Sax Institute, Level 13, Building 10, 235 Jones Street, Ultimo, NSW 2007 Australia; 2 School of Public Health and Community Medicine, University of New South Wales, Sydney, Australia; 3 School of Public Health, University of Sydney, Sydney, Australia; 4 Centre for Healthy Brain Ageing (CHeBA), School of Psychiatry, University of New South Wales, Sydney, Australia

**Keywords:** Health policy, Research, Capacity, Policymaker, Knowledge translation, Evidence

## Abstract

**Background:**

Much has been written about the use of evidence in policy; however, there is still little known about whether and how research is engaged with and used in policy development or the impact of reported barriers and facilitators. This paper aims to (1) describe the characteristics of 131 policy documents, (2) describe the ways in which research was engaged with (e.g. was searched for, appraised or generated) and used (e.g. to clarify understanding, persuade others or inform a policy) in the development of these policy documents, and (3) identify the most commonly reported barriers and facilitators and describe their association with research engagement and use.

**Methods:**

Six health policy and program development agencies based in Sydney, Australia, contributed four recently finalised policy documents for consideration over six measurement periods. Structured, qualitative interviews were conducted with the policymakers most heavily involved in developing each of the 131 policy documents. Interviews covered whether and how research was engaged with and used in the development of the policy product and any barriers or facilitators related to this. Interviews were scored using the empirically validated SAGE tool and thematically analysed. Descriptive statistics were calculated for all key variables and comparisons made between agencies. Multiple regression analyses were used to estimate the impact of specific barriers and facilitators on research engagement and use.

**Results:**

Our data shows large variations between policy agencies in the types of policy documents produced and the characteristics of these documents. Nevertheless, research engagement and use was generally moderate across agencies. A number of barriers and facilitators to research use were identified. No barriers were significantly associated with any aspects of research engagement or use. Access to consultants and relationships with researchers were both associated with increased research engagement but not use. Thus, access to consultants and relationships with researchers may increase the extent and quality of the evidence considered in policy development.

**Conclusions:**

Our findings suggest that those wishing to develop interventions and programs designed to improve the use of evidence in policy agencies might usefully target increasing access to consultants and relationships with researchers in order to increase the extent and quality of the research considered, but that a greater consideration of context might be required to develop strategies to increase evidence use.

**Electronic supplementary material:**

The online version of this article (10.1186/s13012-019-0886-2) contains supplementary material, which is available to authorized users.

## Background

Internationally, many governments have identified increasing the use of evidence from research in policy as an important means of enhancing outcomes and optimising resource allocation [1–4]. As a result, much has been written about whether and how evidence is used in policy formation [5–7] and some knowledge exchange and government agencies have developed resources designed to assist policymakers in using research-based knowledge in their work [8–14]. While a relatively small body of work has begun to explore the extent to which evidence is used in policy development or whether and how specific pieces or bodies of research are taken up [15–17], much of the research in this area remains conceptual [18] or restricted to an examination of policymakers’ beliefs and attitudes regarding the use of evidence [15–17, 19]. There is currently insufficient empirical information available [20] to draw strong conclusions about what might work to increase the use of research evidence in policymaking [19, 21].

Despite the growing number of studies which have sought to examine in detail how evidence is or is not used in the development of specific policies, methodological limitations associated with much of this work means there is still more to be learned [20]. For example, most of the work to date has involved participants being asked to recall their use of evidence in the development of policy in general over a specified period of time (e.g. 5 years [22]). The lack of specificity in this approach is likely to impact on the accuracy of recall [23, 24] and may also obscure important complexities inherent in the use of evidence in policy. Some studies have attempted to use objective measures of research use [25], for example, Zardo and Collie and Bunn et al. identify research cited within specific policy documents [7, 15]. However, this method does not account for the contribution of uncited research which may have played a part, conceptual or otherwise, in the development of the policy. A third approach has been to ask participants to rate the extent to which they drew on a range of evidence sources in developing specific policies; however, information on the ways in which each evidence type was used or the type of influence it had was not elicited [26].

In addition to the gaps in our understanding about whether research is used, there is also much to be learned about the type and extent of research use in policy. While some have called for ethnographic research to examine the research use process in detail [18], little research of this nature has been conducted. Of those studies which use structured measures to collect data, the majority have defined research use very broadly or chosen to focus on a single type of research [7, 26–29]. In studies where more than one type of research use is explicitly considered, imposed use, where research is used in the development of a policy or program at the request or instruction of the developing agency, is rarely included [30, 31]. Another methodological factor limiting our current understanding of research use in policy concerns the relatively slight level of detail collected by most measures. Most measure each type of research use using one [22] or a handful of items [27–29, 31], and they generally risk mono-method bias [32] due to a reliance on a single methodology (e.g. self-report scales) instead of a combination of methods [26].

This lack of detailed analysis of the way in which evidence is used in the development of specific policies and programs means that while much has been written about the barriers and facilitators to research use [19–21], we still know little about how important these factors are in practice and whether and how they impact on evidence engagement or use [21]. This information is required in order to develop the nuanced understanding of evidence use in policy that is needed to underpin effective measurement, interventions, and tools in this space.

**Fig. 1:**
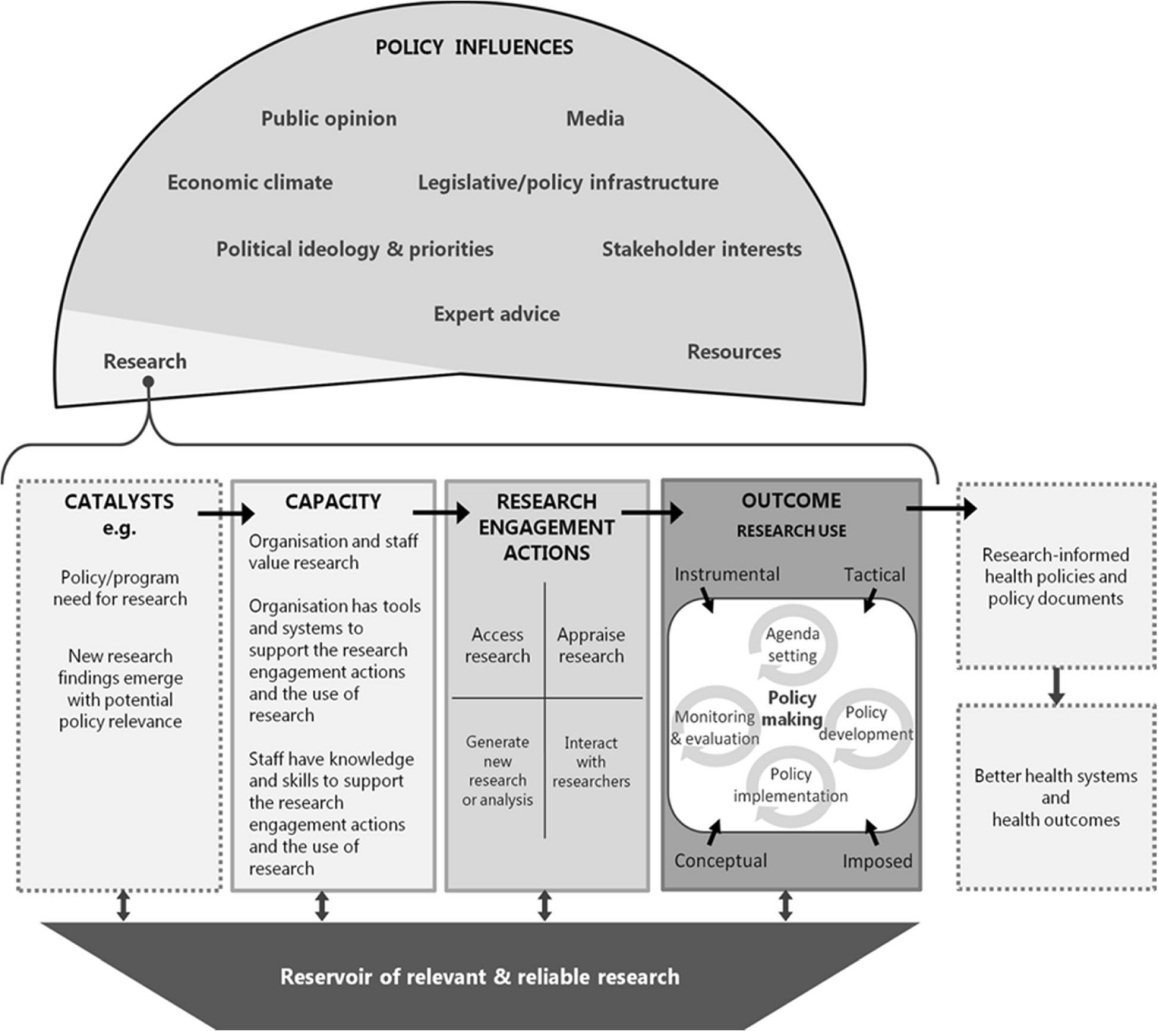
The SPIRIT Action Framework

The exploration of research use in the creation of specific policy products outlined in this paper is framed by the SPIRIT Action Framework (Fig. 1) [33]. The Framework hypothesises that research is just one of many factors which impact on policy decision-making. A catalyst is seen to be required to trigger the use of research, but in order for the trigger to be effective, agencies must have the capacity to engage with research. Where the level of engagement is sufficient, research use may then occur. A necessary condition for effective research engagement and use is a reservoir of relevant and reliable research; however, it should be noted that in many instances, such a reservoir is not available [34, 35].

Research engagement actions are considered to include (1) searching for and (2) obtaining research, (3) appraising its relevance to the policy issue and (4) its quality in terms of methodological rigour and validity, (5) generating new research and/or data analyses, and (6) interacting with researchers [33]. According to the Framework, if the policymaker performs one or more of these actions, and relevant research is obtained, this research can then be used in four different ways in policymaking: (1) instrumental use whereby research evidence directly informs policy [15, 36, 37], (2) conceptual use where research is used to clarify understanding about the policy issue [38–40], (3) tactical use where research evidence is used to help justify and/or persuade others to support a predetermined decision [40, 41], or (4) imposed use where research evidence is used due to legislative, funding, or organisational requirements [42].

This study reports on the way evidence from research was used in the development of 131 policy documents. The policy documents were produced by six agencies participating in a trial of approaches to increasing capacity to use research evidence reported elsewhere [36]. To the best of our knowledge, this constitutes the largest body of empirical evidence surrounding whether and how evidence was used in the creation of specific policy documents assembled to date. By eliciting detailed information in regard to specific documents and using a variety of methods (structured, qualitative interviews and document analysis), we aim to extend the current knowledge base on the use of evidence in policy and highlight potential avenues for enhancing evidence use.

Here, we describe our findings in relation to research engagement and use in the development of specific policy documents and their implications. The specific aims of this paper are to:

1. Describe the evidence use culture in each participating agency.
2. Describe the characteristics of the 131 policy documents.
3. Describe the ways in which research was engaged with and used in the development of the specific policy documents.
4. Identify the most commonly reported barriers and facilitators to research engagement.
5. Explore the relationships between reported barriers and facilitators of research use and reported research use.

## Methods

### Design

A mixed methods approach was used to examine whether and how policymakers engaged with and used research in the development of specific policy documents and the range of factors surrounding this.

### Setting

Six health policy agencies located in Sydney, Australia, were the focus in this study [36] (See Table 1 for agency characteristics). All agencies were participants in SPIRIT, a multifaceted, highly tailored intervention designed to improve the capacity of agencies and staff to engage with and use research in their work. Agencies were eligible to participate in SPIRIT provided that (i) a significant proportion of their work was in health policy or program development, (ii) at least 20 of their staff members were involved in policy or program design, development or evaluation, and (iii) they were located in Sydney. The agency recruitment process is reported elsewhere [36]. As has been previously reported [37], of our six participating agencies, five were state-based and one was a national organisation. The federal and state governments in Australia have responsibility for distinct (although occasionally shared) aspects of the health system. Primary care is federally funded and regulated, while tertiary care is co-funded but state-controlled.

**Table 1:** Participating agencies

	Agency 1	Agency 2	Agency 3	Agency 4	Agency 5	Agency 6
Geographic focus of work	New South Wales	New South Wales	Australia	New South Wales	New South Wales	New South Wales
Remit	Public health	Health systems improvement	Specific aspect of healthcare	Health systems improvement	Specific aspect of health	Specific aspect of health
Policy or guideline development	Yes	Yes	Yes	Yes	Yes	No
Development and roll out of programs	Yes	Yes	Yes	Yes	Yes	Yes
Service delivery	No	No	No	No	No	Yes
Data monitoring	Yes	No	Yes	Yes	No	No
Regulatory oversight	No	No	No	No	No	No
Staff composition	Primarily career public servants	Mix of clinicians and public servants	Mix of clinicians and public servants	Mix of clinicians and public servants	Mix of clinicians and public servants	Primarily clinicians, some public servants

Three of the participating agencies conducted work focussed on specific areas of health or health care, while three worked across public health and health systems improvement. Five agencies developed policies and/or guidelines, and all agencies were responsible for developing and implementing programs. Only one agency’s work included the delivery of services, and none had a regulatory oversight function. Half of all participating agencies included a monitoring or surveillance function as part of their work. Of the five state government agencies, four were board-governed statutory organisations that co-reported to the NSW Ministry of Health, and the fifth was a division within the Ministry itself. The national agency reported to a board but was entirely funded by the Federal Department of Health. Thus, all were government funded and subject to fluctuations in state and federal budgets. All had been operating for at least 3 years but were subject to recent or current restructures.

### Procedure

SAGE (Staff Assessment of enGagement with Evidence) [38] data was collected from all agencies at six time points spaced 6 months apart via structured qualitative interview. At each measurement point, an agency-nominated contact person was asked to nominate four policy documents which had been signed off in the past 6 months to be the focus of the SAGE interviews. All documents were required to meet the following broad criteria of a policy document “A review, report, discussion paper, draft or final policy, formal directive, program plan, strategic plan, ministerial brief, budget bid, service agreement, implementation plan, guideline or protocol with a focus on health service or program design, delivery, evaluation or resourcing [39].”

The agency nominated the person who was considered to have played the most significant role in the document’s development to complete the SAGE interview. Four out of six agencies were not able to locate four documents which met criteria at all six measurement points; thus, the total number of documents considered in the current study is 131. All nominated policymakers were emailed an invitation to participate in a SAGE interview along with standard participant information and consent forms. Interviews were conducted by experienced qualitative interviewers over the phone (unless an in-person interview was requested). Interviews ranged from 30 to 60 min in length. Interviews were audio recorded and professionally transcribed.

### Participant characteristics

Health policy or program staff of participating agencies were regarded as eligible to complete SPIRIT measures if they wrote health policy documents or developed health programs; made or contributed significantly to policy decisions about health services, programs, or resourcing; and were employed at a mid-level or higher in their agency.

In order to be eligible to complete a SAGE interview, participants must also have been nominated by their agency Liaison Person as the person with the greatest amount of knowledge about the development process of a nominated policy document.

### Outcome measure

The SAGE interview (Additional file 1) and scoring tool (Additional file 2) has been described in detail elsewhere [38]. It is a measure developed by the Centre for Informing Policy in Health with Evidence from Research which systematically assesses ten domains of research engagement and use derived from the SPIRIT Action Framework described above [33]. The SAGE interview focuses on the research engagement and research use aspects of Framework. It is conducted in relation to a specific policy document which has been signed off in the last 6 months and takes the form of a semi-structured qualitative interview. The interview includes a series of open-ended questions which map to the ten domains in the SPIRIT Action Framework. The interview format allows for in-depth exploration of whether and how research was used in the development of the document and barriers and facilitators to its use. An empirically derived scoring system has been developed for SAGE [40, 41]. The scoring checklist breaks down each of the ten measured domains (six research engagement actions and four types of research use) into the essential features or main actions associated with them (subactions). Each action has an assigned point value based on its importance in facilitating evidence-informed health policymaking. The degree of importance of each subaction was established through conjoint analysis of surveys completed by over 50 local and international experts in knowledge translation [41, 43]. The points for all ticked subactions are summed to give a score of 0–9 for that particular domain (where 0–2.99 indicates limited, 3–5.99 moderate, and 6–9 extensive efforts to engage with or use research). The scoring system for SAGE has demonstrated good reliability and validity [44].

In addition to the items captured by the SAGE scoring system, for the purposes of this paper, all interviews were also examined to collect information on (a) document type (internal strategy, strategic plan, recommendations or guidelines, description of specific program, research report), (b) whether the document was new (an entirely new piece of work) or ‘updated’, (c) whether the participant content of the document related to an area which is highly researched (no, somewhat/some parts of the document did, yes), (d) whether data was collected to inform the document (no; yes, qualitative; yes, quantitative; yes, qualitative and quantitative), and (e) whether the policy was required to align with legislation or the policy of an umbrella agency. Lastly, interview data was analysed thematically to extract explanatory statements around whether and how research was engaged with and used in the development of the documents in question.

### Quantitative analysis

To address aim 2 and describe the types of policy documents provided by each agency, the number and percentage of documents adhering to five key characteristics were calculated. Chi-squared tests were undertaken to determine whether the characteristics of the documents differed significantly between agencies.

We examined the ways in which research was engaged with and used in the development of the policy documents in question (aim 3) by calculating the mean and standard deviation of each agencies’ scores on the ten domains measured by SAGE, averaged across policy documents and rounds. In order to compare scores on the ten domains by agency, a multivariate analysis of variance (MANOVA) was performed with agency (i.e. agencies 1–6) as the independent variable and the ten SAGE domains as separate dependent variables. A significance level of *α* = 0.05 was used on all comparisons. Due to the high number of comparisons, only significant comparisons are reported.

In order to explore barriers and facilitators to research use (aim 4), we calculated the frequency with which each participant-nominated barrier and facilitator (up to three per document) was reported by each agency. Focusing only on documents where barriers or facilitators were reported, chi-squared tests were used to determine if there was a significant difference between agencies in the type and frequency of each barrier or facilitator identified.

To determine whether the type of barrier/facilitator was related to research engagement and use (aim 5), we created separate indicator variables for each barrier and facilitator (i.e. 1 = yes the barrier/facilitator was present; 0 = the barrier/facilitator was not present). This coding strategy allowed us to examine the unique impact of each barrier or facilitator on research engagement actions and research use. We then performed ten separate multiple regressions where the independent variables were the barriers or facilitators and the dependent variables were the scores on six research engagement actions and four research use types.

To determine whether the number of barriers/facilitators per document was associated with research engagement and research use scores, we performed two separate multivariate analyses of covariance (MANCOVAs). In the first, the dependent variables were the six research engagement actions, and in the second, the dependent variables were the four types of research use. The covariate was the number of barriers or facilitators reported per document.

### Qualitative analysis and back coding

Thematic analysis of the 131 SAGE transcripts led to the identification of clear and consistent themes related to evidence use culture in each agency (aim 1). The SAGE tool does not describe the characteristics of the policy documents considered. Thematic analysis, however, revealed several clusters of document characteristics that appeared to be related to research engagement and use. In order to capitalise on our large data set and quantitatively consider these key themes, we reviewed each transcript to extract information related to the following (relevant data was not available for a small number of documents per theme (maximum 4): (1) document type: we found five distinct categories of policy documents: (i) internal organisational strategies (such as strategic plans), (ii) evaluations of agency policies/programs, (iii) clinical guidelines and recommendations (generally to health service providers), (iv) descriptions of agency programs, and (v) research—generally papers on agency work that had been prepared for peer-reviewed journals; (2) document/policy status: (i) new policy or document or (ii) update on previous policy or document; (3) topic relates to a highly researched area: (i) no, the participant believes there is little research relevant to the topic of the document; (ii) somewhat/partially, the participant believes there is a reasonable amount of research on at least some aspects of what the document covers; and (iii) yes; (4) whether data was collected to inform the document: (i) no; (ii) yes, qualitative; (iii) yes, quantitative; and (iv) yes, qualitative and quantitative; and (5) is the policy required to align with legislation or an overarching agency’s policy: (i) yes and (ii) no.

## Results

### Evidence use culture in participating agencies

Qualitative analyses revealed clear differences between agencies in terms of their evidence use culture. Participants from agencies 1, 3, and 5 consistently underlined the centrality of evidence to their agencies work and mission:

Yes, it’s solidly based on evidence, it has to be. Or you do not have a leg to stand on with policy. Your policy advice has to be soundly grounded in the evidence. Agency 1

In contrast, participants from agencies 2 and 4 were more likely to stress the importance of gaining consensus from relevant clinicians to underpin their policies and programs:

I do not know that any - that all of them would have strong evidence behind but it’s really - yeah, they are really at that level of clinician consensus. Agency 4

This preference for consensus-based decisions was sometimes framed as a consequence of insufficient or contradictory research evidence being available to support decision making:

That is the other thing, like how do you actually sift through all that research and find out - because a lot of the stuff out there is not unanimous. Agency 2

It should be noted that participants from the agencies with a consensus focus still appeared to value research evidence. Many felt, however, that in their specific environment, where the work undertaken was highly complex and contextually specific and innovation was prized, relevant evidence was not available. In instances where relevant evidence was considered to be available, it was still generally thought to be less powerful than having the buy-in of clinical opinion leaders in successfully developing and implementing programs.

Agency 6 operated in an environment where most participants believed there was little relevant research evidence and legislative requirements often left little room for the agency to determine the direction of their programs. For this agency, there was less of a culture of evidence use. Where there was room for the agency to choose the shape or direction of the policies or programs discussed, staff from this agency generally reported drawing on models developed by similar agencies interstate or internationally rather than the research literature.

### Characteristics of the submitted documents

Characteristics of the policy documents submitted by each agency are reported in Table 2. Overall, guidelines and recommendations and descriptions of programs were the most frequently submitted type of policy document (35%). For all agencies, the majority of documents submitted were for new documents/policies (78%), as opposed to updates on previous work.

**Table 2:** Characteristics of the 131 documents

	Agency 1 (*n* = 24) *n* (%)	Agency 2 (*n* = 21) *n* (%)	Agency 3 (*n* = 22) *n* (%)	Agency 4 (*n* = 23) *n* (%)	Agency 5 (*n* = 24) *n* (%)	Agency (*n* = 17) *n* (%)	Total (*N* = 131) *n* (%)
Document type:							
Internal strategy	4 (17)	4 (19)	5 (23)	4 (17)	7 (29)	5 (29)	29 (22)
Evaluation	4 (17)	0 (0)	4 (18)	2 (9.1)	0 (0)	3 (18)	13 (9.9)
Recommendations or guidelines	7 (29)	8 (38)	6 (27)	14 (61)	5 (21)	6 (35)	46 (35)
Description of specific program	8 (33)	8 (38)	5 (23)	2 (8.7)	6 (25)	2 (12)	31 (24)
Research report	1 (4.2)	1 (4.8)	2 (9.1)	1 (4.3)	6 (25)	1 (6)	12 (9.2)
New document/policy (not update) (y/n)							
No	6 (25)	8 (38)	0 (0)	4 (17)	5 (21)	5 (29)	28 (21)
Yes	18 (75)	13 (62)	21 (95)	19 (83)	19 (79)	12 (71)	102 (78)
Data not available	0 (0)	0 (0)	1 (4.5)	0 (0)	0 (0)	0 (0)	1 (0.1)
Topic relates to a highly researched area							
No	5 (21)	4 (19)	6 (27)	12 (52)	11 (46)	13 (76)	51 (39)
Somewhat/partially	8 (33)	10 (48)	5 (23)	4 (17)	8 (33)	4 (24)	39 (30)
Yes	10 (42)	6 (29)	10 (45)	7 (30)	5 (21)	0 (0)	38 (29)
Data not available	1 (4.2)	1 (4.8)	1 (4.5)	0 (0)	0 (0)	0 (0)	2 (15)
Data collected to inform the document:							
No	14 (58)	17 (81)	10 (45)	10 (43)	18 (75)	10 (59)	79 (60)
Yes, qualitative data	7 (29)	2 (9.5)	6 (27)	6 (26)	1 (4.2)	0 (0)	22 (17)
Yes, quantitative data	3 (13)	2 (9.5)	3 (14)	7 (30)	4 (17)	5 (29)	24 (18)
Yes, qualitative and quantitative	0 (0)	0 (0)	2 (9.1)	0 (0)	1 (4.2)	0 (0)	3 (2)
Data not available	1 (4.2)	0 (0)	1 (4.5)	0 (0)	0 (0)	2 (12)	4 (3)
Policy must align with legislation or overarching agency’s policy (y/n)							
No	20 (83)	18 (86)	21 (95)	22 (96)	23 (96)	11 (65)	115 (88)
Yes	4 (17)	3 (14)	0 (0)	1 (4.3)	1 (4.2)	6 (35)	15 (11)
Data not available	0 (0)	0 (0)	1 (4.5)	0 (0)	0 (0)	0 (0)	1 (0.1)

For two thirds of documents, policy agencies reported that there was some or a great deal of relevant research evidence available; there was significant variation between agencies regarding the proportion of documents which participants felt related to a highly researched area (Table 2). A special purpose collection of new data such as analyses of administrative data or qualitative interviews was conducted in relation to 40% of the submitted documents.

Across agencies, only a small proportion of the documents required legislative or overarching policy alignment (11%). Most participants interviewed in relation to documents whose content or direction was heavily constrained by legislation reported that this reduced the perceived need for research evidence to support it:

This stuff is much more operationally based, it is much more about legal, a lot of legal issues, and it does not lend itself to that traditional health sort of research. Agency 6

### Describing the extent of research engagement and research use in policy documents

Mean scores and standard deviations for each Research Engagement Action and each Research Use action are displayed in Table 3 for each agency and for the complete sample of documents. Overall participants reported moderate levels of searching for and accessing research, generating new research, and interacting with researchers. The average scores for appraisal of research relevance and quality fell within the range designated as ‘limited’ by the empirically derived SAGE scoring tool. This accords with many participant reports of difficulties they experienced in relation to appraisal:

One of the things that we said, we still lack real guidance in terms of screening. How do we actually screen the quality of the research? Agency 2

**Table 3:** Agencies’ mean scores and standard deviations for each research engagement and research use action

	Searching for research M (SD)	Research obtained M (SD)	Relevance appraisal M (SD)	Quality appraisal M (SD)	Generate new research M (SD)	Interact with researcher M (SD)	Conceptual research use M (SD)	Instrumental research use M (SD)	Tactical research use M (SD)	Imposed research use M (SD)
Agency 1	4.18 (2.08)	4.68 (1.89)	3.55 (1.73)	2.73 (2.15)	6.34 (2.88)	5.70 (3.79)	5.85 (2.23)	5.65 (2.64)	7.22 (2.98)	4.28 (3.55)
Agency 2	4.79 (1.97)	4.52 (2.01)	3.88 (1.82)	2.72 (1.88)	3.39 (3.30)	5.17 (3.59)	5.35 (2.63)	5.06 (2.97)	7.38 (3.13)	3.17 (2.50)
Agency 3	5.01 (1.91)	5.40 (2.23)	4.03 (2.12)	3.74 (2.08)	5.71 (3.34)	5.15 (4.34)	5.65 (2.16)	6.53 (2.46)	6.83 (3.33)	5.08 (3.20)
Agency 4	5.30 (2.02)	5.13 (1.76)	3.51 (2.02)	2.85 (2.07)	5.60 (2.84)	5.94 (4.16)	5.48 (2.08)	5.71 (2.20)	6.90 (2.49)	3.55 (2.78)
Agency 5	5.35 (1.62)	4.89 (1.86)	4.08 (2.02)	4.64 (1.83)	4.70 (3.31)	5.64 (3.72)	6.26 (1.96)	7.19 (2.03)	7.08 (2.32)	5.15 (3.00)
Agency 6	3.67 (2.02)	3.67 (2.04)	3.34 (1.91)	1.82 (1.44)	4.63 (3.61)	2.22 (3.32)	4.77 (2.89)	4.39 (2.10	5.81 (4.14)	2.06 (2.95)
Total	4.76 (1.99)*	4.76 (2.00)	3.75 (1.92)	3.15 (2.10)*	5.09 (3.28)*	5.10 (3.95)*	5.60 (2.31)	5.83 (2.54)*	6.91 (3.03)	3.97 (3.15)*

indicates a statistically significant difference between agencies on the relevant aspect of research engagement or use

When working in areas in which very little research is available, policymakers noted that usual rules about assessing quality or relevance no longer applied:

The thing is because the research was so limited it’s hard to apply those formal criteria to this particular area of work. Really, because it is so limited, any research is relevant in a way. That’s the approach we took. Agency 5

The reported levels of conceptual and instrumental research use met SAGE scoring tool criteria for ‘moderate use’ on average. The average research use scores were highest for tactical research use, which fell just short of meeting SAGE scoring tool criteria for extensive research use.

To get a consensus and progress the policy, we had to really use research - the most up to date research we could and base it on that. Or otherwise we would still be in the steering committee, teleconferences, debating around in circles. Agency 2

Imposed research use was the least common type, with the average score falling within the upper range of limited research use. Those who reported imposed research use tended to frame it more in terms of a strong cultural assumption than a specific directive:

They do require us to, but I think it’s more because anything that we need to do within this area needs to be evidence-based and we need to be providing clinicians with the most updated information and the most updated evidence to support any sort of work or initiatives that they need to do. Agency 2

The analysis revealed a significant multivariate main effect of agency: *F*(50, 590) = 1.535, *p* = 0.013, and partial *η*^2^ = 0.115. To explore this effect, we examined univariate tests for each of the dependent variables separately (Table 3). There was a significant effect of agency on the following SAGE domains: searching for research, quality appraisal, generating new research, interactions with researchers, instrumental research use, and imposed research use.

### Barriers to and facilitators of evidence use in each agency, and the relationship to research engagement and use

#### Barriers

Table 4 reports the frequency of the most commonly reported barriers to research use reported by participants. No barriers to research use were reported for 19% of the documents submitted. The most frequently reported barrier to research use was not having enough time (25%). While interviewees reported that some of the documents presented had been completed over a reasonably long time period, many others were reported to have been completed in a matter of days or weeks. These tight timelines gave little room for research reviews and findings to be sought:

I think certainly a lot of the guys that I work with here have the expertise to really pick apart a piece of research, determine whether it’s relevant and of high quality, but there’s not always the time, and sometimes that kind of approach is not necessarily supported by management, because if we did that for every task that we have, we just would not get through the workload. You know? Agency 1

**Table 4:** Number of documents reporting each type of barrier per agency*

	None *n* (% of agency docs barrier reported for)	Time *n* (% of agency docs barrier reported for)	Own skills *n* (% of agency docs barrier reported for)	Lack of evidence *n* (% of agency docs barrier reported for)	Poor access to literature *n* (% of agency docs barrier reported for)	Other *n* (% of agency docs barrier reported for)
Agency 1 (*n* = 24 docs)	6 (25)	12 (50)	1 (4.2)	7 (29)	1 (4.2)	3 (13)
Agency 2 (*n* = 21 docs)	6 (29)	6 (29)	8 (38)	3 (14)	4 (19)	3 (14)
Agency 3 (*n* = 22 docs)	2 (9.1)	5 (23)	3 (14)	6 (27)	9 (41)	3 (14)
Agency 4 (*n* = 23 docs)	3 (13)	9 (39)	5 (22)	6 (26)	7 (30)	0 (0)
Agency 5 (*n* = 24 docs)	7 (29)	6 (25)	2 (8.3)	5 (22)	11 (48)	0 (0)
Agency 6 (*n* = 17)	8 (47)	5 (29)	1 (5.9)	3 (18)	2 (12)	2 (12)

*Note that the number of reported barriers exceeds the number of documents because multiple barriers (up to 3) could be recorded for individual documents

The next most commonly reported barrier was having poor access to research literature (e.g. paid subscriptions to research journals and databases) (20%):

..there were some (journal articles) that we just could not get hold of, and so it was just the abstracts that I was able to use and nothing more, just because we did not have the access to it, or they needed to be paid for, or you had to have a subscription to actually access it. Agency 2

A perceived lack of relevant research evidence was also common (18%):

It’s interesting when you are trying to develop projects in this kind of space because when you are looking at an evidence-based kind of paper, it might be a trial but it might only be a really small cohort of people. Whereas we are wanting to look at something that we can institute state-wide. So what might have worked in a very controlled way in a specific district, you know, in the UK, may not actually be applicable to New South Wales context. Agency 5

Focusing only on policy documents where barriers were reported (i.e. *N =* 98), chi-squared tests revealed a significant relationship between agency and the type of barriers reported, *χ*^2^(20, *N =* 139) = 32.95, *p* = 0.03. There was, however, no significant difference between agencies in the proportion of documents for which the lack of evidence was a reported barrier or between agency and the number of barriers reported per document.

We next explored whether the number of barriers per document was associated with research engagement and research use scores. The multivariate tests for both research engagement actions, *F*(6, 122) = 1.19, *p = .*32, and *η*_*p*_^2^ = 0.06, and research use, *F* < 1, were not significant, indicating that there was no association between the number of barriers reported per document and the scores on research engagement actions and research use.

Lastly, we explored whether the type of barrier was related to research engagement and use. The multiple regression models for each of the SAGE domains were nonsignificant (all *ps > 0.05*), indicating that the type of barrier was not a significant predictor of research engagement and use.

#### Facilitators

Table 5 reports the frequency of the nine most commonly reported types of facilitators to research use by agency. Across the 131 policy documents, 165 facilitators were reported.

**Table 5:** Number of documents reporting each type of facilitator per agency*

	None	Consultants	Internal research use expertise	Journal access through uni	Relationships with researchers	Advisory group	High-quality evidence	Internal data	Agency library or journal access	Other
Agency 1 (*n* = 24 documents)	3 (13)	8 (33)	8 (33)	4 (17)	5 (21)	3 (13)	3 (13)	3 (13)	2 (8.33)	3 (13)
Agency 2 (*n* = 21 documents)	6 (29)	0 (0)	6 (29)	1 (48)	3 (14)	0 (0)	0 (0)	2 (9.5)	6 (29)	2 (9.5)
Agency 3 (*n* = 22 documents)	2 (9.1)	1 (4.5)	10 (45)	0 (0)	5 (23)	3 (14)	0 (0)	1 (4.5)	3 (14)	3 (14)
Agency 4 (*n* = 23 documents)	5 (22)	6 (26)	12 (52)	1 (8.7)	3 (13)	0 (0)	0 (0)	1 (4.3)	6 (26)	2 (8.7)
Agency 5 (*n* = 24 documents)	2 (8.3)	5 (21)	14 (58)	1 (4.2)	3 (13)	4 (17)	3 (13)	2 (8.3)	2 (8.3)	1 (4.2)
Agency 6 (*n* = 17 documents)	5 (29)	0 (0)	5 (29)	3 (18)	0 (0)	1 (5.9)	1 (5.9)	3 (24)	0 (0)	1 (5.9)
Total (*n* = 131 documents)	23 (18)	20 (15)	55 (42)	10 (7.6)	19 (15)	11 (8.4)	7 (5.3)	12 (9.2)	19 (15)	12 (9.2)

*Note that the total number of reported facilitators exceeds the total number of documents because multiple facilitators (up to 3) could be recorded for individual documents

Across all agencies, and within each agency, the most frequently reported facilitator to research use was having internal research use expertise. However, there were variations between agencies in other frequently reported facilitators. In agencies 1 and 4, consultants were the next most frequently reported facilitator. In agencies 4 and 2, access to library/journals/databases was the second most frequently reported facilitator. For agency 3, relationships with researchers were the second most frequently reported facilitator.

We explored whether the number of facilitators per document was associated with research engagement and research use scores. The multivariate test for the research engagement action model was significant, *F*(4, 125) = 4.45, *p* < 0.001, and *η*_*p*_^2^ = 0.18, but not for research use, *F*(4, 125) = 2.03, *p = .*09, and *η*_*p*_^2^ = 0.06. This suggests that facilitators were more strongly related to the research engagement actions as opposed to research use.

## Discussion

The current study represents the largest scale detailed examination of how research was engaged with and used in the development of specific policy documents to date. We found moderate levels of most types of research use and engagement overall, but that these varied according to agency and key document characteristics. A range of barriers and facilitators to research use were reported; however, reported barriers were not significantly associated with levels of research engagement or use. In contrast, access to consultants and relationships with researchers were associated with greater engagement with research evidence but were not significantly associated with evidence use. Our findings reveal some important considerations in both the measurement of research engagement and use and in the targeting of interventions to increase the use of research evidence in policy.

The six agencies participating in the current study had different remits, and these were reflected in their evidence use cultures. The evidence use culture in agencies 1, 3, and 5 was markedly stronger than in the other participating agencies, with participants frequently noting that their work was expected to be evidence-based. This focus on evidence was clear in some of the core functions of these agencies; all of which included monitoring or surveillance of relevant health or health performance data. All of these agencies also funded and conducted their own research; indeed, 25% of the documents contributed by agency 5 were research papers. The work of agencies 2 and 4 was centred on health systems improvement. Participants in these agencies stressed the key role of innovation and the development of new models and strategies in their work and reported that only a third of their documents related to highly researched areas. For participants from these agencies, obtaining clinician consensus was generally seen to be a more powerful predictor of success in the development and roll out of their programs than was research evidence. As has been noted elsewhere, this was particularly so in areas where the available evidence was highly contested [34]. Agency 6 was unique amongst our sample in that part of their remit involved the delivery of health services and policy and/or guideline development was not a key feature of their work. Participants in this agency were notably less likely than others to report searching for research evidence, often due to the belief that there was no relevant evidence available. Reported levels of imposed research use were notably low for participants from this agency. Instead, the staff at the agency tended to look to the work of similar bodies interstate or overseas to inform their activities. The potential pitfalls associated with this approach have been widely discussed in the literature (e.g. [45]).

When the SAGE interview and scoring tool were developed, we chose to adopt a broad definition of a ‘policy product’. There was no data on which to base assumptions about which if any of these document types might be more common, or on whether these or any other document characteristics would vary by agency. Amongst our sample of health policy agencies in New South Wales, guidelines and documents which described a policy or program were the most common document types submitted but there was a considerable variation between agencies in the proportion of submitted documents sitting within each category. This accords with the distinction Head notes in the literature between the chief functional roles played by different agencies and the variation in information needs which arise from this [34]. As the use of research evidence is virtually mandatory in some of these document types (e.g. research documents) but may far more discretionary in others (e.g. internal strategies), it may be useful for future studies to take a narrower definition of a policy product or to compare like documents with like. Indeed, others [11, 15] have noted variations in evidence use amongst different types of policy documents. Adopting a narrower definition of a policy product, however, would likely necessitate that agencies produce a larger number of documents than was required in our study, however, and in the case of most of our participating agencies at least, would not have been possible. It is also noteworthy that nearly a fifth of the documents submitted were updates of previous policies, guidelines, or reports. In some instances, there may be a less perceived need for evidence review when updating a policy or program as opposed to creating a new one.

Another key document characteristic that varied significantly between agencies was the extent to which the participant responsible for developing the document considered that it related to a well-researched area. For half of the participating agencies, around half of their documents were judged by participants to relate to an area which was not well researched. Head [34] has previously noted that little research evidence is available in relation to some policy areas. This appeared to be particularly true for agency 6, with three quarters of their documents reported to relate to an under-researched area. While these categorisations are based on subjective assessments, they seem reasonable given that a lack of relevant research to guide policy decisions has been documented in numerous studies [46, 47].

The potential impact of working in an area in which little research evidence is available is illustrated by agency 6, for which this problem was particularly prevalent, and which tended to score significantly lower than most agencies on many aspects of research engagement and use. If an agency works primarily in an area in which little evidence exists, it would seem likely that this places a ceiling on the extent to which they can engage with and use research that is challenging to move significantly regardless of any capacity building efforts to the contrary. This raises questions regarding how to make fair comparisons between agencies when the richness of evidence available to them may be vastly different [34]. It also underscores the importance of the sometimes-overlooked flipside of the evidence-informed policy coin and the need for researchers to provide timely and relevant research to fulfil policy needs [48]. Of note, the potential for agency 6 to use evidence was likely further impacted by the relatively high proportion (one third) of documents they submitted that were reportedly required to align with legislation or the policy of an overarching agency. Although others have reported evidence being used to justify or gain support for mandated approaches [48]), it is perhaps not surprising that where the policy content is pre-determined, an agency may not prioritise resources to researching issues related to it. Thus, while agency 6 received low scores on research engagement and use, it appeared that engaging with evidence was actually quite often not considered when developing policy documents. This was reflected in the fact that participants from this agency were also the least likely to report any barriers to having used research evidence. These findings underline the important role contextual factors play in the extent to which policy agencies engage with and use evidence and how they may help to both create and perpetuate different evidence use cultures. Tailored approach are likely to be an important feature of successful attempts made to increase the use of evidence.

Overall participants reported moderate levels of searching for and accessing research, generating new research, and interacting with researchers (the different ways of accessing evidence, types of evidence sought, and how they are scored in SAGE are listed in Additional file 2). The average scores for appraisal of research relevance and quality fell within the limited range, consistent with comments from many participants about their perceived lack of skill in relation to evidence appraisal and previous reports of such skill deficits representing a key barrier [21]. The reported levels of conceptual and instrumental research use were moderate on average, as was tactical research use, which fell just short of meeting criteria for extensive. While tactical research use is sometimes positioned as a negative use of research, in our study this did not appear to be the case. Rather, this appeared to be a positive finding with participants reporting that it was becoming increasingly necessary to demonstrate that research evidence underpinned proposed policies or programs if they were to be approved by their managers, advisory committees, and the like. Wye et al. [48] also noted that research was often used to persuade or justify a course of action, to uphold public accountability, and to ensure agencies were able to withstand potential challenges from internal and external sources.

Imposed research use was the least common research use type in our study, with the average score falling within the upper range of limited. We are not aware of any other studies which have measured imposed research use, so we cannot comment as to whether this is a typical pattern of results; however, Wye et al. [48] reported that amongst participants from the agencies they studied, there was considered to be an expectation that they brought a research perspective to their role and were ‘on top’ of the evidence in their area of work. It has been suggested that low levels of imposed research use may reflect a limited agency culture around the use of evidence [20]. Overall, this appeared to be less the case here with participants reporting that, while they were not explicitly asked or required to use evidence, they considered the need to use evidence to be assumed.

Barriers and facilitators to research use in policy have now been explored in several studies (e.g. [19–21]. This paper extends on what is currently known by (a) exploring barriers and facilitators to research use in relation to particular policy documents and (b) illuminating not just what the barriers and facilitators were reported to be, but also what impact they had on research engagement and use. The latter has been previously noted as a major gap in the existing literature [1]. No barriers to research use were reported for nearly a fifth of documents. In keeping with the literature to date, not having enough time to adequately consider the available research evidence was the most commonly reported barrier [16, 21, 48]. The second most prevalent barrier, poor access to literature (e.g. paid subscriptions to research journals and databases) (20%), has also been noted previously [21, 47–49]. Clearly, despite increases in the availability of research evidence due to open access journals and research repositories like Health Systems Evidence (https://www.healthsystemsevidence.org/), there is still a need for further increases. To this end, Kitson et al. [50] have recommended that research funding organisations require open access publication as a key means of supporting knowledge translation. While different barriers were more or less common at different agencies, the number of barriers experienced on average varied little. Further, while participants often expressed frustration with perceived barriers to research use, their impact appears to have been negligible in practice. Indeed, the agency that was least likely to report experiencing any barriers also reported the lowest level of evidence use. Overall, we found no association between either the type or the number of barriers reported per document and scores on research engagement or research use.

The most frequently reported facilitator to research use overall was having internal research use expertise, the importance of this factor has been noted previously [16, 21, 51, 52]. Harnessing external expertise in the form of paid consultants was the second most common facilitator for two agencies. Paying consultants to synthesise evidence appears to be relatively common amongst our sample, but this may not be the case elsewhere (although some government agencies internationally have trialled or implemented into routine practice the establishment of partnerships with researchers that include evidence review/briefing services, e.g. [35, 53, 54]). Our findings show that access to consultants was a unique predictor of the number and types of research accessed, the extent to which evidence was appraised for relevance and quality and interactions with researchers. This suggests that higher quality evidence was likely to be found when consultants were engaged. Engagement of consultants was not associated with the extent of research use, however. This accords with a body of research which suggests that access to evidence alone is not sufficient to increase the use of research in policy. For example, Wilson et al. concluded that receiving on-demand access to an evidence briefing service amongst six NHS Clinical Commissioning Groups in England [35] did not result in significantly increased intentions to use research evidence in their work, while Van Egmond’s depiction of the multifaceted, carefully constructed system the Centre VTV has developed in order to produce policy-relevant evidence for the Dutch government suggests that the process of providing useable evidence to policy may extend far beyond simply synthesising the available evidence [54].

Relationships with researchers, the second most commonly nominated facilitator for agency 3, was a unique predictor of the number and types of research accessed, and (understandably) interactions with researchers, but was not related to extent of research use. This finding accords with those of other studies which have noted that in the fluid, fast-moving world of policymakers, conversations with researchers provide an efficient means of obtaining information quickly [42, 48] and often appear to be preferred even when formal evidence briefing services are available [35]. As Wye [48] points out, however, the information imparted through conversations with researchers is highly dependent on the researcher in question and may not always reflect the best available evidence. It appears that overall access to consultants and relationships with researchers likely increased the quality of the evidence used, if not the extent to which it was used. This is an encouraging finding for those seeking to improve the use of evidence in policy. Given that we know that research evidence will rarely if ever be the primary basis of policies and programs, increasing the quality of the evidence that is considered, even if not directly used, may be the most impactful change that can be made in this space. Our findings suggest that focusing on key facilitators, access to consultants and relationships with researchers, may be the most effective way of promoting higher quality research engagement. Overall though, while gleaning some useful information in relation to research engagement, the lack of relationship noted between reported barriers and facilitators and actual research use provides some support for the suggestion that in failing to properly account for contextual factors, the barriers and facilitators approach may inadequately capture the key determinants of evidence use [18, 55, 56]. In our own work, we found a multilevel, highly tailored research use intervention (SPIRIT [36]) which included a focus on contextual factors (such as the extent to which research was valued at all levels of the agency and the systems and structures in place to support its use) to be effective in increasing capacity to use research at both a staff and an agency level [57].

The current study makes a significant contribution to the literature in presenting detailed information on how research was engaged with and used, using a validated measure, and in relation to a large number of specific policy documents. Another major strength of this study is its novel contribution in quantitatively measuring the relationship between barriers and facilitators to research use and actual research engagement and use. A need for information of this kind has been previously noted [21]. Nonetheless, our decision to present much of our data quantitatively means the resultant study does not grapple with the full complexity of evidence use in policy to the extent which may have been possible had an in-depth qualitative analysis of the data been undertaken instead. A limitation of the comparisons between agencies in our study is that while some agencies produced a relatively large number of policy documents and were able to choose those which they felt best represented their use of evidence to be included in the study, others struggled to locate even four to be assessed at each time point. Thus, we are comparing some agencies’ best with the full range of other agency’s work.

In this study, we measured the barriers and facilitators participants reported regarding their use of research evidence in assembling the policy document in question and made a novel contribution by quantifying how these related to research engagement and actual research use. This approach reflects our pragmatic approach as researchers with an interest in intervention, and of our participating agencies which were looking to increase their use of evidence, in seeking concrete strategies to guide change. As noted above, however, the usefulness of this approach has been questioned by some authors for its failure to fully capture context [18, 55, 56]. We acknowledge that this is a limitation of our study also (while the prompt for the relevant questions did ask about contextual factors, they were rarely mentioned by participants). A deeper exploration of context is likely to be a useful addition to future work in this area. A further critique of the barrier and facilitator approach is that it tends to focus on the use of *research* evidence, when in fact policy makers value a whole range of evidence types. While SAGE collects information on all types of evidence accessed, these are collated as part of the scoring system and have not been presented separately here. It was beyond the scope of this paper to include a detailed analysis of the types of evidence agencies engaged with.

While the current study is the first and largest of its kind, our findings, drawn as they are from our sample of six health policy agencies in New South Wales Australia, all of whom had chosen to participate in an intervention designed to increase their capacity to use evidence, may not be broadly generalizable. Further, our data is derived from self-report and thus is subjective. We are unable to make objective assessments of the extent to which research was used in policy documents and or on the process by which research evidence was or was not used.

## Conclusion

In conclusion, our data shows large variations between policy agencies in the types of policy documents produced and the characteristics of these documents. Nevertheless, research engagement and use was generally moderate across agencies. Our findings suggest promising directions for those wishing to develop interventions and programs designed to improve engagement with evidence in policy agencies, namely increasing access to consultants and relationships with researchers in order to increase the quality of the evidence used. They also suggest a greater focus on agency context might be the next critical step in identifying strategies for increasing evidence use.

Contributions to the literature

- There is currently little evidence to guide efforts to increase the use of evidence in policy.
- Our large-scale examination of how research was engaged with and used in the development of specific policy documents highlights the diversity of research use cultures and contexts in policy agencies, and how these relate to agency remit.
- Findings add to the literature by revealing no association between reported research use barriers and actual research use in policy and highlighting access to consultants and researchers as useful strategies for increasing the quality of the evidence used in policy

## Additional files

Additional file 1: SAGE: Assessing the use of research in policy products—interviewer’s guide. (PDF 250 kb)

Additional file 2: SAGE scoring tool. (PDF 184 kb)

## Abbreviations

CIPHER: Centre for Informing Policy in Health with Evidence from Research
SAGE: Staff Assessment of enGagement with Evidence from research
SPIRIT: Supporting Policy In health with Research: an Intervention Trial

## References

[1] Cabinet Office (1999). Modernising government, white paper.

[2] HM Government (2013). What works: evidence centres for social policy. In: Office C, editor.

[3] Rudd K (2008). Address to heads of agencies and members of senior executive service.

[4] World Health Organization (2004). World report on knowledge for better health: strengthening health systems.

[5] Elliott H, Popay J (2000). How are policy makers using evidence? Models of research utilisation and local NHS policy making. J Epidemiol Community Health.

[6] Greenhalgh T, Russell J (2009). Evidence-based policymaking: a critique. Perspect Biol Med.

[7] Zardo P, Collie A (2014). External factors affecting decision-making and use of evidence in an Australian public health policy environment. Soc Sci Med.

[8] Campbell D, Donald B, Moore G, Frew D (2011). Evidence check: knowledge brokering to commission research reviews for policy. Evid Policy.

[9] Canadian Foundation for Health Care Improvement. Executive Training for healthcare improvement (EXTRA). Available from: http://www.cfhi-fcass.ca/WhatWeDo/EducationandTraining/EXTRA.aspx. Last accessed April 2019.

[10] Canadian Institute of Health Research. Best Brains Exchanges. Available from: http://www.cihr-irsc.gc.ca/e/43978.html. Last accessed April 2019.

[11] Dobbins M, Cockerill R, Barnsley J, Ciliska D (2001). Factors of the innovation, organization, environment, and individual that predict the influence five systematic reviews had on public health decisions. Int J Technol Assess Health Care.

[12] Dobbins M, Hanna SE, Ciliska D, Manske S, Cameron R, Mercer SL (2009). A randomized controlled trial evaluating the impact of knowledge translation and exchange strategies. Implement Sci.

[13] The Cochrane Collaboration. The Australasian Cochrane Centre. Policy Liaison Initiative. Available from: http://www.cochrane.org.au/projects/policy.php. Last accessed April 2019.

[14] Sax Institute. Health Policy & Research Exchange. Available from: https://www.saxinstitute.org.au/policy-makers/find-a-researcher/. Last accessed April 2019.

[15] Bunn F, Kendall S (2011). Does nursing research impact on policy? A case study of health visiting research and UK health policy. J Res Nurs.

[16] Weatherley H, Drummond M, Smith D (2002). Using evidence in the development of local health policies: some evidence from the United Kingdom. Int J Technol Assess Health Care.

[17] Aaserud M, Lewin S, Innvaer S, Paulsen EJ, Dahlgren AT, Trommald M (2005). Translating research into policy and practice in developing countries: a case study of magnesium sulphate for pre-eclampsia. BMC Health Serv Res.

[18] Oliver K, Lorenc T, Innvær S (2014). New directions in evidence-based policy research: a critical analysis of the literature. Health Res Policy Syst.

[19] Innvaer S, Vist G, Trommald M, Oxman A (2002). Health policy-makers’ perceptions of their use of evidence: a systematic review. J Health Serv Res Policy.

[20] Orton L, Lloyd-Williams F, Taylor-Robinson D, O'Flaherty M, Capewell S (2011). The use of research evidence in public health decision making processes: systematic review. PLoS One.

[21] Oliver K, Innvar S, Lorenc T, Woodman J, Thomas J (2014). A systematic review of barriers to and facilitators of the use of evidence by policymakers. BMC Health Serv Res.

[22] Amara N, Ouimet M, Landry R (2004). New evidence on instrumental, conceptual, and symbolic utilization of university research in government agencies. Sci Commun.

[23] Wattenmaker WD, Shoben EJ (1987). Context and the recallability of concrete and abstract sentences. J Exp Psychol Learn Mem Cogn.

[24] Walker I, Hulme C (1999). Concrete words are easier to recall than abstract words: evidence for a semantic contribution to short-term serial recall. J Exp Psychol Learn Mem Cogn.

[25] Innvaer S (2009). The use of evidence in public governmental reports on health policy: an analysis of Norwegian official report (NOU). BMC Health Serv Res.

[26] Armstrong R, Waters E, Dobbins M, Anderson L, Moore L, Petticrew P (2013). Knowledge translation strategies to improve the use of evidence in public health decision making in local government: intervention design and implementation plan. Implement Sci.

[27] Landry R, Amara N, Lamari M (2001). Utilization of social science research knowledge in Canada. Res Policy.

[28] Landry R, Amara N, Lamari M (2001). Climbing the ladder of research utilization. Sci Commun.

[29] Landry R, Lamari M, Amara N (2003). The extent and determinants of the utilization of university research in government agencies. Public Adm Rev.

[30] Hanney S, Gonzalez-Block M, Buxton M, Kogan M (2003). The utilisation of health research in policy-making: concepts, examples and methods of assessment. Health Res Policy Syst.

[31] de Goede J, van Bon-Martens MJ, Putters K, van Oers HA (2012). Looking for interaction: quantitative measurement of research utilization by Dutch local health officials. Health Res Policy Syst.

[32] Cook TD, Campbell DT (1979). Quasi-experimentation: design and analysis issues for field settings.

[33] Redman S, Turner T, Davies H, Haynes A, Williamson A, Milat A (2015). The SPIRIT Action Framework: a structured approach to selecting and testing strategies to increase the use of research in policy. Soc Sci Med.

[34] Head BW (2015). Towards more ‘evidence-Informed’ policy making?. Public Adm Rev.

[35] Wilson PM, Farley K, Bickerdike L, Booth A, Chambers D, Lambert M (2017). Does access to a demand-led evidence briefing service improve uptake anD use of research evidence by health service commissioners? A controlled before and after study. Implem Sci.

[36] The CIPHER Investigators (2014). Supporting Policy In health with Research: an Intervention Trial (SPIRIT)—protocol for a stepped wedge trial. BMJ Open.

[37] Makkar S, Williamson A, Turner T, Louviere J, Redman S, Green S (2016). ORACLe: a measure of an organisation’s capacity to engage in evidence-informed health policy. Health Res Policy Syst.

[38] Makkar SR, Brennan S, Williamson A, Turner T, Redman S, Green S (2015). The development of SAGE: a tool to evaluate how policymakers’ engage with and use research in health policymaking. Res Eval.

[39] Haynes A, Turner T, Redman S, Milat AJ, Moore G (2015). Developing definitions for a knowledge exchange intervention in health policy and program agencies: reflections on process and value. Int J Soc Res Methodol.

[40] Makkar S, Williamson A, Turner T, Redman S, Louviere J (2015). Using conjoint analysis to develop a system to score policymakers’ engagement with research in policy and program development. Health Res Policy Syst.

[41] Makkar SR, Williamson A, Turner T, Redman S, Louviere J (2015). Using conjoint analysis to develop a system to score research engagement actions by health decision makers. Health Res Policy Syst.

[42] Haynes A, Gillespie JA, Derrick GE, Hall WD, Redman S, Chapman S (2011). Galvanizers, guides, champions, and shields: the many ways that policymakers use public health researchers. Milbank Q.

[43] Makkar SR, Williamson A, Turner T, Redman S, Louviere J (2015). Using conjoint analysis to develop a system of scoring policymakers' use of research in policy and program development. Health Res Policy Syst.

[44] Makkar SR, Williamson A, D'Este C, Redman S (2017). Preliminary testing of the reliability and feasibility of SAGE: a system to measure and score engagement with, and use of research in health policies and programs. Implem Sci.

[45] Shipan CR, Volden C (2012). Policy diffusion: seven lessons for scholars and practitioners. Public Adm Rev.

[46] Barwick MA, Boydell KM, Stasiulis E, Ferguson HB, Blase K, Fixsen D (2008). Research utilization among children’s mental health providers. Implem Sci.

[47] Huckel Schneider C, Campbell D, Milat AJ, Haynes A, Quinn E (2014). What are the key organisational capabilities that facilitate research use in public health policy?. Public Health Res Pract.

[48] Wye L, Brangan E, Cameron A, Gabbay J, Keling JH, Pope C (2015). Evidence based policy making and the ‘art’ of commissioning - how English healthcare commissioners access and use information and academic research in ‘real life’ decision-making: an empirical qualitative study. BMC Health Serv Res.

[49] Wye L, Brangan E, Cameron A, Gabbay J, Klein JH, Anthwal R (2015). What do external consultants from private and not-for-profit companies offer healthcare commissioners? A qualitative study of knowledge exchange. BMJ Open.

[50] Kitson A, Bisby M. Speeding up the spread: putting KT research into practice and developing an integrated KT collaborative research agenda: Alberta Heritage Foundation for Medical Research; 2008. Available from: http://cihc.ca/files/members/pke/SpeedingUpTheSpread_KT08.pdf

[51] Jewell CJ, Bero LA (2008). “Developing good taste in evidence”: facilitators of and hindrances to evidence-informed health policymaking in state government. Milbank Q.

[52] Green A, Bennett S (2007). Sound choices: enhancing capacity for evidence-informed health policy.

[53] Heller DJ, Hoffman C, Bindman AB (2014). Supporting the needs of state health policy makers through university partnerships. J Health Polit Policy Law.

[54] Egmond SV, Bekker M, Bal R, Grinten TVD (2011). Connecting evidence and policy: bringing researchers and policy makers together for effective evidence-based health policy in the Netherlands: a case study. Evid Policy.

[55] Davies HTO, Powell AE, Nutley SM (2015). Mobilising knowledge to improve UK health care: learning from other countries and other sectors - a multimethod mapping study. Health Serv Deliv Res.

[56] Ward V, Smith S, House A, Harmer S (2012). Exploring knowledge exchange: a useful framework for practice and policy. Soc Sci Med.

[57] Williamson A, Barker D, Green S, D’Este C, Davies HTO, Jorm L (2019). Increasing the capacity of policy agencies to use research findings: a step wedge trial. Health Policy Res Syst.

